# Review of infective dose, routes of transmission and outcome of COVID-19 caused by the SARS-COV-2: comparison with other respiratory viruses

**DOI:** 10.1017/S0950268821000790

**Published:** 2021-04-14

**Authors:** Sedighe Karimzadeh, Raj Bhopal, Huy Nguyen Tien

**Affiliations:** 1 School of Medicine, Sabzevar University of Medical Sciences, Sabzevar, Iran; 2 Usher Institute, University of Edinburgh, Edinburgh EH3 9AG, UK; 3 School of Tropical Medicine and Global Health, Nagasaki University, Nagasaki, Japan

**Keywords:** COVID-19, infective dose, respiratory viruses, SARS-CoV-2, viral dynamics, viral load

## Abstract

Severe acute respiratory syndrome coronavirus 2 (SARS-CoV-2) is pandemic. Prevention and control strategies require an improved understanding of SARS-CoV-2 dynamics. We did a rapid review of the literature on SARS-CoV-2 viral dynamics with a focus on infective dose. We sought comparisons of SARS-CoV-2 with other respiratory viruses including SARS-CoV-1 and Middle East respiratory syndrome coronavirus. We examined laboratory animal and human studies. The literature on infective dose, transmission and routes of exposure was limited specially in humans, and varying endpoints were used for measurement of infection. Despite variability in animal studies, there was some evidence that increased dose at exposure correlated with higher viral load clinically, and severe symptoms. Higher viral load measures did not reflect coronavirus disease 2019 severity. Aerosol transmission seemed to raise the risk of more severe respiratory complications in animals. An accurate quantitative estimate of the infective dose of SARS-CoV-2 in humans is not currently feasible and needs further research. Our review suggests that it is small, perhaps about 100 particles. Further work is also required on the relationship between routes of transmission, infective dose, co-infection and outcomes.

## Introduction

Coronavirus disease 2019 (COVID-19) is a severe acute respiratory syndrome caused by coronavirus 2 (SARS-CoV-2) which is now pandemic. Several fundamental virologic concepts relating to COVID-19 remain poorly understood such as the initiating event and infective dose i.e. number of particles to cause a detectable infection. For understanding viral pathogenicity, determining the number of particles that trigger infection is crucial. A low infectious dose could mean the organism is highly transmissible person-to-person and via touching contaminated surfaces [1]. Viral load is one of the main aspects of viral kinetics in infectious disease transmission [2]. It can help develop prevention and control strategies and risk models of disease [2]. Little is known on whether viral load correlates with disease severity and progression. Route of exposure to an infectious agent is also important [3]. Different outcomes from no infection to subclinical or clinical infection can be observed after exposure to the infectious agent [3]. It is unclear whether the number of particles on exposure is correlated with the severity and outcome of disease, however for some infectious diseases a dose−response relationship between dose of infectious agent at exposure and outcome of disease has been reported [4]. Understanding of these concepts requires experimental studies to complement epidemiologic data that can provide limited insights into these matters. Improved understanding of viral concepts of SARS-CoV-2 can promote more effective outbreak control strategies. We did a rapid review of the evidence for the infectious dose, viral load, co-infection, route of transmission and correlation with the outcome of SARS-CoV-2 infection. To help interpret the limited data available we compared viral dynamics of SARS-CoV-2 with other respiratory pathogens such as influenza virus, SARS-CoV-1 and Middle East respiratory syndrome (MERS-CoV) viruses.

## Methods

We identified relevant data for this review by searching databases including PubMed and Google Scholar, using the terms ‘Infective dose’, ‘Respiratory viruses’, ‘SARS-CoV’, ‘MERS-CoV’, ‘Aerosol’, ‘COVID-19’, ‘viral load’, ‘Coronavirus’, ‘Influenza virus’. The latest literature search was performed on 1 September 2020 with no restriction on date of publication and study design. We included articles published in English with full-text version available. We did not limit our search to peer-reviewed journals.

## Result

We included 79 experimental and human studies exploring the infective dose, viral load, route of administration, exposure and outcome in respiratory viruses. We extracted data for respiratory viruses including coronaviruses (Seasonal CoV, SARS-CoV-1, SARS-CoV-2, MERS-CoV), influenza virus, rhinovirus, coxsackievirus, adenovirus and respiratory syncytial virus (RSV).

### Infective dose

The main methods for defining the infective viral dose is through studies utilising dilution of virus studies for cytopathogenic effect (CPE) in 50% of inoculated culture cells (known as tissue culture infectious dose, or TCID_50_), or by counting plaque-forming units; each plaque in a layer of host cells indicating colonisation by a single virus particle (plaque forming unit − PFU) [5]. TCID_50_ is the viral dose that induces either pathological changes or cell death in 50% of inoculated tissue cultures. The viral plaque assay is a quantitative measure of the number of particles that form a plaque, estimating viral concentration in plaque-forming units [6]. A virus titre of 0.7 PFU can be estimated as theoretically equivalent to 1 TCID_50_, so given that most studies reported the latter we converted the results for those reporting PFU [6]. For determining the infectious dose (ID_50_) in humans the viral administration should, ideally, be in controlled experiments. Since patient safety concerns would usually make this unethical, animal-based experimental studies are mostly used for simulating infection in humans [7]. We have summarised in Tables 1 and 2 the infectious dose reported for some major human respiratory viruses identified by either experimental infection in human volunteers or laboratory animals.

**Table 1.** Infective dose of relevant respiratory viruses in humans

Virus	Strain	Dose		Route of administration	References
		TCID_50_	PFU		
^a^Coronavirus	HCoV-229E	13	9	NR	Watanabe [8]
^b^Influenza	H1N1	1.0 × 10^3^	700	IN	Hayden [9]
	H2N2	0.6–3	0.42–2.1	Aerosol	Alford [10]
	H3N2	1.0 × 10^7^	7 000 000	IN	Treanor [11]
^c^Rhinovirus	RV15	0.032	0.0224	IN	Couch [12]
^d^Adenovirus	Type 4	0.5	0.35	Aerosol	Couch [13]
^e^Coxsackievirus	A21-48654	6	4.2	IN	Couch [12]
^f^RSV	Ts-1	30–40 (33% infected)	21–28	IN	Parrott [14]
	Type 39	100	70	Aerosol	Bischoff [15]

TCID50; %50 tissue infective culture dose, PFU; plaque-forming units, RSV; respiratory syncytial virus, NR; not reported, IN; intranasal.

**Table 2.** Experimental studies on the infective dose of coronaviruses in various mammals

Virus	Host	Dose (PFU)	Route of inoculation	Numbers and/or %, signs of infection	References
SARS-CoV-2	^a^Ferret	221 359	IN	6/6	Kim [16]
		500	IN	16.7,1/6	Ryan [17]
		50 000	IN	6/6	
		5 000 000	IN	6/6	
		420 000	IN	4/4	Richard [18]
SARS-CoV-2	^b^hACE2 mice	70 000	IN	36.8,7/19	Bao [19]
HCoV-OC43		400 000	IN	3/3	Sun [20]
		4 000 000	IG	1/3	
		630	Aerosol	2/2	Bao [21]
		21 000	IN	50% Lethal	Jiang [22]
		100 000	IN	40% Lethal	Dinnon [23]
	BALB/c mice	16 000	IN	3/3	Gu [24]
	BALB/c and C57B6 mice	70 000	IP/IC	100% Lethal	Jacomy [25]
	BALB/c mice	70	IC	100% Lethal	Shen [26]
SARS-CoV-1	tgMice	280	IN	NR	Watanabe [8]
MERS-CoV	tgMice	0.7	IN	NR	Tao [27]
		7	IN	50% Lethal	
SARS-CoV-2	^c^Cynomolgus macaques	48 600	Aerosol	4/4	Johnston [28]
		700 000	IN/IT	4/4	Rockx [29]

Virus	Host	Dose (PFU)	Route of inoculation	Numbers and/or %, signs of infection	References
SARS-CoV-2	^d^Rhesus macaques	700 000	IO	2/2	Deng [30]
			IT	1/1	
			IG	0/2	
		1 820 000	IN/IT/IO/Oral	8/8	Munster [31]
		11 000	IN/IT	3/3	Chandrashekar [32]
		110 000	IN/IT	3/3	
		1 100 000	IN/IT	3/3	Johanston [28]
		28 700	Aerosol	4/4	
SARS-CoV-2	^e^African green monkeys	38 000	Aerosol	3/3	Johanston [28]
		500 000	IN/IT	6/6	Woolsey [33]
		3 000 000	IN	6/6	Cross [34]
		2000	Aerosol	2/2	Blair [35]
		3 610 000	IO/IT/IN/Oral	2/2	
SARS-CoV-2	^f^Syrian hamster	398 107	IN/IO	4/4	Imai [36]
		1000	IN/IO	4/4	
		56 000	IN	3/3	Sia [37]
		100 000	IN	75,24/36	Osterrieder [38]
	Immunocompromised syrian hamster	100	IN	10/10	Brocato [39]
		1000	IN		
		10 000	IN	40% Lethal	
	^g^Bats	70 000	IN	78,7/9	Schlottau [40]

TCID_50_, %50 tissue infective culture dose; PFU, plaque-forming units; tgMice, transgenic mice; hACE2, human angiotensin converting enzyme 2, BALB/c; begg albino laboratory-bred mouse, IN; intranasal, IG; intragastric, IO; intraocular, IT; intrathecal, IC; intracerebral, IP; intraperitoneal, NR; not reported.

### Human studies on infective dose of SARS-CoV-2 and other relevant viruses

Irrespective of the route of inoculation, some respiratory viruses such as rhinoviruses and adenoviruses mostly cause asymptomatic or mild respiratory symptoms in immunocompetent hosts. Influenza is one of the most contagious and rapidly spreading viruses with a very low infective dose [41]. Although other factors are important in transmission of the virus, the minimum infective dose of SARS-CoV-2 causing COVID-19 in humans is unknown it is assumed to be low since the virus transmits rapidly and is more contagious [42, 43]. The route of inoculation affects the response to viruses [7]. Infective dose assessment in human studies requires intranasal administration of the virus via drops or aerosols. Infection with drops informs us about upper respiratory tract infection, while aerosols can inform about lower respiratory tract infection [7].

We found no experimental studies of this kind in humans but observational studies. Nasopharyngeal and endotracheal samples of SARS-CoV-2 infected patients showed no growth after eight days post incubation in Vero cells. Median tissue culture infective dose was calculated as 1780 TCID50/ml [44]. Isolation of SARS-CoV-2 from oropharyngeal and nasopharyngeal sample of one patient in the USA and inoculation in Vero cells shows that SARS-CoV-2 can replicate rapidly and achieve 10^5^ TCID_50_/mL within 24-hour post-infection [45] (study not tabulated). Although virus titre peaked at >10^6^ TCID_50_/ml after 48 h post-inoculation, major CPE (cytopathogenic effect) was observed after 60 h post-inoculation [45]. This infective dose is much higher than rhinovirus but lower than for influenza virus and similar to coxsackievirus when administered nasally.

Table 1 shows human studies in healthy volunteers on other relevant respiratory viruses.

- Coronavirus

The human ID_50_ for seasonal coronavirus subtype 229E that causes mild common cold in humans was reported to be 13 TCID_50_ [8].

1. Influenza

The infective dose for H1N1 strain of influenza virus by nasal drop was 10^3^ TCID_50_ (Table 1, B) [9]. For the H2N2 strain by aerosol administration that TCID_50_ was 0.6–3.0 TCID_50_ [10], higher than by intranasal drop (127–320 TCID_50_) [46]. For the H3N2 strain by nasal drop was 1 × 10^7^ TCID_50_ [11].

1. Rhinovirus

The TCID_50_ of rhinovirus when administered by aerosols at 0.68 TCID_50_ was about 20 times greater than by nasal drops (0.032 TCID_50_) [12].

1. Adenovirus

For Adenovirus type 4 the TCID_50_ was 35 TCID_50_ by intranasal route and 0.5 TCID_50_ by aerosol [13]. In this study 6.6 particles by aerosol (corresponding to 462 particles by nasal drop) were required to initiate infection in 50% of the population. Furthermore, a high dose of virus by nasal drops was found to cause infection in the lower intestinal tract [13].

1. Coxsackievirus

TCID_50_ of coxsackievirus A21 strain was 6 TCID_50_ when administered by intranasal droplet compared with 28–34 TCID_50_ by aerosol [12].

1. Respiratory syncytial virus (RSV)

Attenuated vaccine strain of RSV, TS-1, at a dose of 30–40 TCID_50_ infected infants. This infectious dose of RSV is assumed to be lower than with the wild strain because of its lesser virulence through multiple passages in tissue culture [14]. Type-39 had a TCID_50_ of 100 [15].

### Animal studies

#### SARS-CoV-2

Table 2 summarises experimental animal studies on SARS-CoV-2.

- Ferret

Intranasal inoculation of 10^5.5^TCID_50_ (221 359 PFU) of SARS-CoV-2 virus presented raised body temperature and decreased activity in ferrets [16]. One out of six ferrets that were infected by intranasal route at a dose of 500 PFU showed signs of upper respiratory tract viral replication. Meanwhile, all ferrets presented with pulmonary histopathological features and viral RNA replication at higher doses (50 000– 5 000 000 PFU) [17, 18].

1. Mice

An study on human angiotensin converting enzyme 2 (hACE2) transgenic mice after intranasal inoculation at a dose of 10^5^ TCID_50_ (70 000 PFU) of SARS-CoV-2 showed weight loss and viral replication in the lungs [19]. Another study on both young and aged hACE2 mice after infection at a dose of 400 000 PFU (≈5.71 × 10^5^ TCID_50_) by intranasal route showed mild weight loss (10%) and more severe histopathological features of interstitial pneumonia in aged mice [20]. Mice infected by the intragastric route at a dose of 4 000 000 PFU (≈5.71 × 10^6^ TCID_50_) showed pulmonary infection in one of three mice [20]. Transgenic mice after aerosol inoculation of SARS-CoV-2 isolates at a dose of 630 PFU showed viral RNA, interstitial pneumonia and pulmonary infiltration after at least 25 min exposure to the virus [21]. After intranasal infection with 21 000 PFU of SARS-CoV-2, three out of six hACE2 mice died at 6 days post infection [22]. Similarly 40% mortality in begg albino laboratory-bred (BALB/c) mice was observed after intranasal infection with SARS-CoV-2 at a dose of 100 000 PFU [23]. BALB/c mice showed viral replication and interstitial pneumonia at a dose of 16 000 PFU by the intranasal route [24].

1. Cynomolgus macaques

After aerosol inoculation at a dose of 48 600 PFU macaques presented modest clinical signs, viral RNA and pulmonary pathological features [28]. After inoculation at a dose of 700 000 PFU (10^6^ TCID_50_) of SARS-CoV-2 intranasally and intrathecally, cynomolgus macaques presented no clinical signs, however, histopathological changes indicating diffuse alveolar damage and viral replication were observed [29].

1. Rhesus macaques

Rhesus macaques infected with SARS-CoV-2 at a dose of 700 000 PFU (10^6^ TCID_50_) via ocular conjunctivae presented mild pneumonia and higher viral RNA than those infected intrathecally, whereas no viral RNA was detected after exposure by the intragastric route [30]. After inoculation at a dose of 2 600 000 TCID_50_ (1 820 000 PFU) of SARS-CoV-2 by the intranasal, intratracheal, oral and ocular routes, macaques showed various range of clinical signs including weight loss, piloerection, decreased appetite, pallor and dehydration [31]. Exposure to higher doses and correlation with signs of infection such as decrease in appetite and response to stimuli as well as slight neutropenia and lymphopenia was observed in a group of rhesus macaques that were infected at a dose of 1 100 000 PFU (≈1.57 × 10^6^ TCID_50_). Two groups of rhesus macaques that were infected by intranasal and intrathecal route at a dose of 110 000 PFU (≈1.57 × 10^5^ TCID_50_) and 110 000 PFU (≈1.57 × 10^4^ TCID_50_) presented mild clinical disease. Histopathological features of pneumonia were observed at a dose of 110 000 PFU [32]. Rhesus macaques exposed by aerosol route at a dose of 28 700 PFU showed mild clinical signs of pulmonary infection [28].

1. African green monkey

All three African green monkeys exposed to 36 000 PFU by the aerosol route showed clinical signs of pulmonary disease [28]. African green monkeys inoculated by combined intranasal and intrathecal routes at a dose of 500 000 PFU (≈7.14 × 10^5^ TCID_50_) showed histopathological features of pulmonary lesions and no overt clinical signs of disease [33]. At a dose of 3 000 000 PFU (≈4.28 × 10^6^ TCID_50_) they showed efficient viral replication and respiratory signs of infection [34]. Two African green monkeys exposed at a dose of 2000 PFU by the aerosol route and 3 610 000 PFU by combined route of intranasal, thecal, ocular and oral showed signs of acute respiratory distress syndrome (ARDS), increased level of interleukin 6 (IL6) and cytokine storm [35].

1. Hamsters

In two groups of juvenile and adult hamsters infected by intranasal and ocular routes with SARS-CoV-2 at a higher and lower dose of 10^5.6^ PFU (≈5.68 × 10^5^ TCID_50_) and 1000 PFU (≈1.42 × 10^3^ TCID_50_), respectively, higher dose infected hamsters presented more severe lung complications, earlier weight loss and earlier pneumomediastinum than the lower dose group [36]. Hamsters that were intranasally inoculated at a dose of 56 000 PFU showed weight loss and viral shedding [37]. After intranasal infection at a dose of 100 000 PFU hamsters showed both clinical presentation and viral RNA [38]. Immunosuppressed hamsters after intranasal inoculation at doses of 100 and 1000 PFU showed extreme weight loss whereas death was observed in those exposed to 10 000 PFU [39].

1. Bats and other animals

Intranasal inoculation of 10^5^ TCID_50_ (70 000 PFU) of SARS-CoV-2 isolates into fruit bats, pigs, chickens, cats, dogs (data not tabulated for the latter four species) showed no clinical signs and viral RNA replication in except slight viral RNA and shedding in cats and bats [40, 47].

#### Other coronaviruses

We examined findings on other coronaviruses, including seasonal CoV, SARS-CoV-1 and MERS-CoV for relevant insights. Two groups of BALB/c mice and c57 black strain 6 (C57BL/6) mice after infection with HCoV-OC43 at a dose of 10^5^ TCID_50_ (70 000 PFU) by intraperitoneal and intracerebral route showed 100% lethality at 8 days [25]. However, at a dose of 10^4^–10^5^ TCID_50_ (7000– 70 000 PFU) they presented no clinical signs and viral RNA by intraoral route and mild signs of infection by intranasal route at 21 days postnatal [25]. In another study, 12-day-old BALB/c mice exposed by the intracerebral route at a dose of 100 TCID_50_ (70 PFU) of wild-type HCoV-OC43 showed 100% lethality 4 days later [26]. Estimated infectivity of SARS-CoV-1 was comparable to other coronaviruses including HCoV-229E, a causative agent for a mild cold in humans. ID_10_ and ID_50_ of SARS-CoV-1 were reported as 43 and 280 PFU (400 TCID_50_) in an experimental study [8]. A study on transgenic mice reported the ID_50_ of MERS-CoV as <1 TCID_50_ and LD_50_ as 10 TCID_50_ [27]. Transgenic mice that were infected with MERS by the intranasal route presented signs of infection at a dose between 100 and 500 000 PFU (≈142 and ≈7.14 × 10^5^ TCID_50_) [48, 49].

### Transmission route, exposure rate and correlation with outcome

SARS-CoV-2 transmission is thought to be mainly through respiratory droplets and fomites rather than through aerosols carried over long distances [50]. There are questions about whether the size of the infectious dose of SARS-CoV-2 and its route of transmission correlates with disease severity.

SARS-CoV-2 was not thought to be transmitted long distances by an aerosol in 75 465 COVID-19 patients in China [51]. A study on aerosol distribution of SARS-CoV-2 in Wuhan hospital reported the maximum distance of transmission as 4 m in hospital wards. Reflecting this, an increased risk of positivity at sampling site and objects observed in patients' treatment areas (40.6%) than office areas of physicians (12.5%) [52].

SARS-CoV-1 transmission is thought to be increased by 20.4-fold when people have at least exposure for >30 min and distance of <1 m with infected patients [53]. However, a safer physical distance to avoid transmission of SARS-CoV-2 is 1 m as recommended by WHO and approximately as 2 m by CDC [54, 55]. Small droplets can, nonetheless, be found at a distance of 7–8 m away [56]. The rate of SARS-CoV-2 transmission was increased by an estimated 18.7-fold in an enclosed area compared with the outdoor environment [57]. Transmission of SARS-CoV-2 via contaminated surfaces or aerosolisation was observed in cluster analysis of COVID-19 patients [58].

During the SARS-CoV-1 outbreak in 2003 the higher risk of infection was correlated with the amount and setting of exposure [53]. In the Amoy-Garden housing complex in Hong Kong, the lower concentrations of the virus explained the lower risk of infection in the upper floors [59]. It was estimated that the apartment's residents were exposed to 16–160 PFU (≈22.8–228 TCID_50_) per person depending on the floor [8].

Given the absence of direct information about SARS-CoV-2, findings from other respiratory viruses and in animals may provide clues. The potential of airborne, aerosol transmission of SARS-CoV-2 was observed in ferrets and cats [16, 60]. Aerosol inoculation with the H3N2 strain of sub-lethal influenza virus in laboratory mice, presented exacerbated mortality and morbidity, pulmonary infiltration and inflammation, as well as 6-fold higher levels of IL-6 expression in the lungs compared to intranasally inoculated mice [61]. Consistently, African green monkeys infected by the aerosol route of SARS-CoV-2 (Table 2) presented with ARDS, increased level of IL6, and cytokine storms [35].

Increased exposure to the influenza virus, presumably reflecting increased infective dose, was correlated with disease progression [62]. In addition to studies of SARS-CoV-2 infected ferrets, rhesus macaques and hamsters [17, 32, 36, 39] studies on laboratory adapted mice infected with HCoV-OC43, SARS-CoV-1 and MERS-CoV reported increased morbidity and lethality with increasing dose at exposure [8, 48, 49].

### SARS-CoV-2 viral load and outcome

COVID-19 has lower morbidity and mortality, but greater infectivity, compared with SARS and MERS [63]. The serial interval, the duration of the symptoms between the onset of symptoms in an index case and the secondary case, of COVID-19 together with viral shedding results suggest much transmission occurs early, even before onset of symptoms [64, 65]. This interval is about 3 days for influenza virus [66], 4 days for SARS-CoV-2 [64], 8.4 days for SARS-CoV-1 [67] and 14.6 days for MERS-CoV [68]. This means that infected people with SARS-CoV-2 and influenza can spread the virus faster than SARS-CoV-1 and MERS-CoV. Most COVID-19 studies show the highest viral load before or at and shortly after the onset of symptoms [65, 69–71], which may account for the rapid spreading of disease [72, 73]. The high viral load in throat swabs at or just before onset of symptoms suggests that 44% of transmission can occur in the asymptomatic stages [70]. SARS-CoV-2 and influenza virus share a similar pattern of viral shedding [29, 65]. There is correlation between higher viral load and the severity of COVID-19 [74, 75]. Patients with severe symptoms of COVID-19 in one study presented 60 times higher viral load and prolonged viral shedding than patients with mild symptoms [76]. In another study higher viral load was not correlated with outcomes including ICU admission, mortality and oxygen requirement in hospitalised patients [77]. In a study on 4172 patients, higher viral loads were observed in the first phase of the outbreak and the first phase of disease. The same study reported lower viral loads in ICU patients than patients in other wards [78].

A similar viral load was observed among different age groups in one study [78] while another study found a higher viral load in children aged <5 years than adults [79]. The viral loads in asymptomatic patients were similar to those in patients with mild-to-moderate COVID-19 [65]. Prolonged viral shedding, initial high viral load and increased risk of transmission in the early stage of disease was also observed in patients with seasonal coronavirus (OC43 and 229E) [80]. Patients with single seasonal coronavirus had a higher viral load than patients with co-infection [81]. Children with high viral loads of seasonal coronavirus were found to have an increased risk of symptomatic infection [80].

Studies on hamsters and African green monkeys reported no correlation between viral load and initial exposure dose of SARS-CoV-2 [35, 36] and SARS-CoV-1 [36, 82]. In contrast viral load and inoculating dose were associated in laboratory mice that were infected with SARS-CoV-1 [83] respiratory syncytial virus (RSV) [84] and influenza virus [85].

During the SARS-CoV-1 outbreak at Amoy Gardens complex higher viral loads were detected in residents living in units adjacent to the index case indicating a link with exposure dose [86]. Inter-study and inter-species variability highlight that correlation of viral load and dose at exposure is not unequivocal.

## Discussion

Effective prevention and control strategies in the pandemic of COVID-19 require understanding of infective dose, transmission and coinfection. We found limited evidence on these points requiring us to examine the data for other relevant viruses and to combine observations on animals and humans. In humans (Table 1) the infective dose varies greatly by virus and route of administration. However, for coronavirus and influenza, mostly hundreds or even more virus particles are required to cause an infection. Similarly, in animals (Table 2) the infective dose varies greatly by species and by route of administration. Our review may help in selection of best animal models for experimental studies. The infective dose is generally large, with hundreds and even millions of virus particles being required to induce disease. We estimate that the infective dose for SARS-CoV-2 is probably lower than for influenza virus (1000 TCID_50_) as it is more contagious with a slightly higher R0. The only human study on a coronavirus we found was on HCoV-229E with the TCID_50_ comparison was 13 [8]. The infective dose in humans for SARS-CoV-2 was estimated as 100 particles based on computational analysis of nasopharynx in transmission and inhalation of droplets [87]. In animals the minimum dose of SARS-CoV-2 that infected immunocompromised hamsters were also 100 particles [39], whereas healthy ferrets and transgenic mice were infected at slightly higher dose of 500 particle by nasal [17] and 630 particles by aerosol route [21]. Possibly the higher value of 100 particle can be used as a potential surrogate for estimating the minimum infective dose of SARS-CoV-2 in humans.

The findings of this study are relevant to the science underlying the use of respiratory mask in preventing transmission of viral particles [88]. The data summarised here and in Table 2 shows that the true measurement of infectious dose in animals and extrapolation to human is not possible. First, none of the animal studies reported the same clinical presentations and pathology after infection with SARS-CoV-2, and outcomes were highly variable as in humans. Second, different endpoints used for the measurement of infection, meanwhile susceptibility of animals can largely vary dependent on various species, ACE2 expression, age and comorbidities. Third, the route of inoculation can largely affect the response of animals to infection. All the animals infected by aerosol and other routes of exposure presented signs of infection whereas animals exposed by the intragastric route mostly remained asymptomatic (intranasal route being intermediate). In animals, the infective dose is generally lower with aerosol transmission than other routes. The infective dose in human could be lower than currently believed if transmission by aerosol is important. Moreover, aerosol transmission can allow the virus to penetrate into the lower respiratory tract of humans and cause severe symptoms [7].

The route of infection can impact on the induction of innate and adaptive immune responses [61]. Little is known about the host immune response following different routes of infection with SARS-CoV-2. Higher viral load is not necessarily correlated with more severe symptoms, with some studies finding higher viral load in mildly symptomatic or asymptomatic stages of disease [72, 77, 78]. This suggests a decline in viral load as the disease progresses [77, 78].

COVID-19 shares important features with influenza in serial interval of disease, clinical presentation, transmission route, viral load, infective dose, viral shedding and correlation with outcome. Studies on influenza virus suggest a correlation between increasing body mass index (BMI) and increased aerosol shedding through increased frequency of small airway closure and reopening [89]. High BMI is associated with critical illness and severity of symptoms in patients with COVID-19 and influenza [90, 91].

Exhaled breath of symptomatic patients with influenza can transmit an estimated 33 particles per minute in aerosol [89]. Twenty minutes of exposure would be required for the exposure to the median infective dose of H1N1 subtype. Similarly, almost 25 particles per minute (630 particles in 25 min) in aerosol were required to cause SARS-CoV-2 infection in hACE2 mice [21]. Exposure for a similar period to SARS-CoV-2 exhaled in normal breathing of infected patients could lead to the inhaling of our estimated hundreds of SARS-CoV-2 particles by aerosol, thus complementing infection by fomites and droplets. However, further studies are warranted to examine infective dose by the aerosol route and its correlation with COVID-19 severity and immune response both in animals through experiments and humans through observation.

## Conclusion

SARS-CoV-2 has distinct features as well as commonalities compared with other similar respiratory pathogens justifying further experimental and observational studies concentrating on transmission, exposure, the infective dose, viral load, virus shedding and the synergistic effect of viral dose and route of exposure and co-infection of SARS-CoV-2 with one or more respiratory pathogens. This review has merely laid the foundation in the study of this topic which is important for the development of rational public health strategies to minimise spread of infection.

## Data Availability

The authors confirm that the data supporting the findings of this study are available within the article.
